# Cross-Neutralizing Antibodies to Pandemic 2009 H1N1 and Recent Seasonal H1N1 Influenza A Strains Influenced by a Mutation in Hemagglutinin Subunit 2

**DOI:** 10.1371/journal.ppat.1002081

**Published:** 2011-06-09

**Authors:** Wei Wang, Christine M. Anderson, Christopher J. De Feo, Min Zhuang, Hong Yang, Russell Vassell, Hang Xie, Zhiping Ye, Dorothy Scott, Carol D. Weiss

**Affiliations:** 1 Laboratory of Immunoregulation, Division of Viral Products, Center for Biologics Evaluation and Research, US Food and Drug Administration, Bethesda, Maryland, United States of America; 2 Division of Hematology, Center for Biologics Evaluation and Research, US Food and Drug Administration, Bethesda, Maryland, United States of America; 3 Office of Biostatistics and Epidemiology, Center for Biologics Evaluation and Research, US Food and Drug Administration, Bethesda, Maryland, United States of America; 4 Laboratory of Pediatric and Respiratory Diseases, Division of Viral Products, Center for Biologics Evaluation and Research, US Food and Drug Administration, Bethesda, Maryland, United States of America; Johns Hopkins University - Bloomberg School of Public Health, United States of America

## Abstract

Pandemic 2009 H1N1 influenza A virus (2009 H1N1) differs from H1N1 strains that circulated in the past 50 years, but resembles the A/New Jersey/1976 H1N1 strain used in the 1976 swine influenza vaccine. We investigated whether sera from persons immunized with the 1976 swine influenza or recent seasonal influenza vaccines, or both, neutralize 2009 H1N1. Using retroviral pseudovirions bearing hemagglutinins on their surface (HA-pseudotypes), we found that 77% of the sera collected in 1976 after immunization with the A/New Jersey/1976 H1N1 swine influenza vaccine neutralized 2009 H1N1. Forty five percent also neutralized A/New Caledonia/20/1999 H1N1, a strain used in seasonal influenza vaccines during the 2000/01–2006/07 seasons. Among adults aged 48–64 who received the swine influenza vaccine in 1976 and recent seasonal influenza vaccines during the 2004/05–2008/09 seasons, 83% had sera that neutralized 2009 H1N1. However, 68% of age-matched subjects who received the same seasonal influenza vaccines, but did not receive the 1976 swine influenza vaccine, also had sera that neutralized 2009 H1N1. Sera from both 1976 and contemporary cohorts frequently had cross-neutralizing antibodies to 2009 H1N1 and A/New Caledonia/20/1999 that mapped to hemagglutinin subunit 2 (HA2). A conservative mutation in HA2 corresponding to a residue in the A/Solomon Islands/3/2006 and A/Brisbane/59/2007 H1N1 strains that circulated in the 2006/07 and 2007/08 influenza seasons, respectively, abrogated this neutralization. These findings highlight a cross-neutralization determinant influenced by a point mutation in HA2 and suggest that HA2 may be evolving under direct or indirect immune pressure.

## Author Summary

Influenza A viruses mutate to escape neutralization by antibodies. These mutations predominantly occur in the globular head of the hemagglutinin protein, while the stalk is more conserved. Pandemic 2009 H1N1 influenza virus differs from seasonal H1N1 strains that circulated in the past 50 years and resembles a strain that did not circulate but was used in the 1976 swine influenza vaccine. We investigated whether persons immunized with either the 1976 swine influenza or recent seasonal influenza vaccines, or both, have antibodies that cross-neutralize pandemic 2009 H1N1. Sera from 1976 swine influenza vaccine trials cross-neutralized pandemic 2009 H1N1 and to a lesser extent the A/New Caledonia/20/1999 H1N1 strain that was used in vaccines during the 2000/01–2006/07 influenza seasons. Sera from persons who received several seasonal influenza vaccines containing A/New Caledonia/20/1999 H1N1 cross-neutralized pandemic 2009 H1N1, regardless of whether they received the 1976 swine influenza vaccine. We found that cross-neutralization between 2009 H1N1 and A/New Caledonia/20/1999 frequently mapped to the hemagglutinin stalk. A mutation in the stalk of strains circulating during the 2007/08–2008/09 seasons abrogates this neutralization. These findings highlight a cross-neutralization determinant influenced by a point mutation in the hemagglutinin stalk and suggest that the stalk may be evolving under direct or indirect immune pressure.

## Introduction

**Figure 1:**
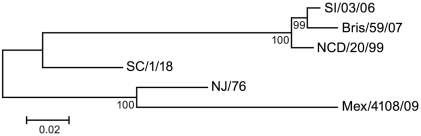
Genetic relationships among H1N1 HA. A phylogenetic tree was constructed for HA from six H1N1 influenza A strains, including recent seasonal strains, 2009 H1N1, and historic 1976 and 1918 influenza A strains. SI/03/06: A/Solomon Islands/3/2006; Bris/59/07: A/Brisbane/59/2007; NCD/20/99: A/New Caledonia/20/1999; SC/1/18: A/South Carolina/1/1918; NJ/76: A/New Jersey/1976; Mex/4108/09: A/Mexico/4108/2009.

In June 2009 the World Health Organization declared a new influenza pandemic due to sustained human to human transmission in several geographic regions of the novel swine-origin influenza A H1N1 virus, which was first identified in April by the Centers for Disease Control and Prevention (CDC) of the United States of America [1]. This novel H1N1 virus, referred to as pandemic 2009 H1N1 virus (2009 H1N1), has a hemagglutinin (HA) of classical swine lineage viruses that have circulated in the swine population for decades with little change in HA antigenicity [2]. The 2009 H1N1 HA is antigenically different from those of recent human seasonal influenza H1N1 viruses, but is closely related to A/New Jersey/1976 (NJ/76) influenza virus (Figure 1), a strain used in 1976 to immunize approximately 45 million people in the US during the swine influenza vaccination campaign after a localized outbreak [3]. However, NJ/76 influenza virus did not circulate. Emergence of the novel pandemic 2009 H1N1 virus raised questions about whether immunization with the 1976 swine or recent seasonal influenza vaccines could confer any protection. Several groups have reported that older persons may have substantial cross-immunity to the 2009 H1N1, though the literature is mixed on the degree of cross-immunity induced by prior seasonal influenza vaccines [4]–[9].

Influenza virus surface glycoprotein HA mediates virus entry and is the most important target of antibody-mediated protection. Cellular proteases cleave the HA precursor (HA0) to generate the HA1 surface subunit that mediates the binding to cell surface sialic acid receptors and the HA2 transmembrane subunit that mediates membrane fusion between viral and endosomal membranes after endocytosis (reviewed in [10], [11]). During infection and vaccination, HA elicits neutralizing antibodies. Antigenic maps of HA show that HA1 is the major target of neutralizing antibodies that inhibit virus binding to target cells [12], [13] and are classically detected by the hemagglutination inhibition (HI) assay. However, HA2 is more conserved than HA1. Neutralizing antibodies that bind to the stalk region of HA2 have been shown to confer broadly cross-neutralizing activity against several subtypes of viruses across clades but within a group [14]–[20] and to provide protection in animal models [16], [18]–[20]. These antibodies typically do not have HI activity and appear to neutralize virus by interfering with HA-mediated conformational changes required for virus entry [14], [17], [18].

Using lentiviral pseudovirions bearing HA on their surface (HA-pseudotypes) [21], we investigated whether persons immunized in 1976 with the NJ/76 swine influenza vaccine or more recently with seasonal influenza vaccines produced neutralizing antibodies to 2009 H1N1. Both sera from the 1976 swine influenza vaccine trials and contemporary sera from a cohort of subjects who received recent seasonal influenza vaccines, regardless of whether they received the 1976 swine influenza vaccine or not, often contained cross-neutralizing activity to 2009 H1N1. Some of this cross-neutralizing activity was dependent on the HA2 subunit and surprisingly was sensitive to a naturally-occurring variant at position 89 in HA2 that emerged in recent years. The implications of these findings for potential immune escape are discussed.

## Results

### Vaccination against A/New Jersey/1976 and neutralizing antisera to 2009 H1N1

**Figure 2:**
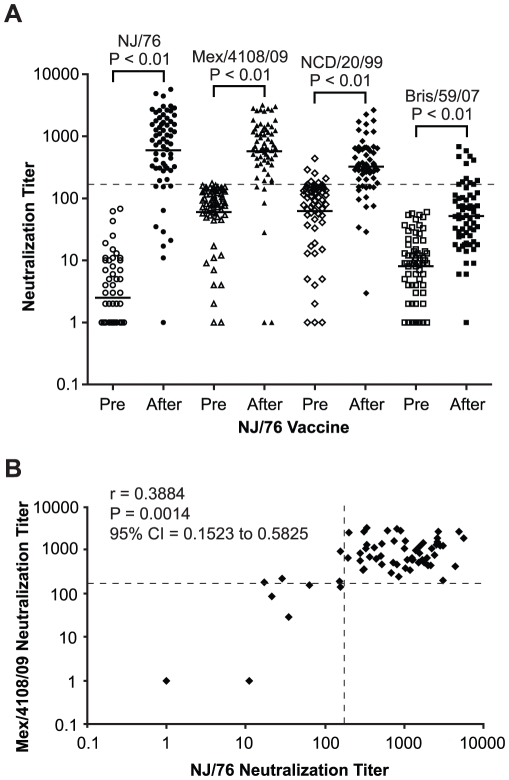
NJ/76 (swine flu) vaccination generates cross-neutralizing antibodies to 2009 H1N1 and seasonal influenza NCD/20/99. (A) Neutralization titers to NJ/76, Mex/4108/09, NCD/20/99 and Bris/59/07 in NJ/76 clinical trial sera collected pre and post NJ/76 vaccination are shown. The geometric mean of titers (GMT) of neutralization in each group is indicated by the short line. P values were calculated by the comparison of pre and post (after) NJ/76 vaccination with paired t test. (B) Correlation between NJ/76 and Mex/4108/09 neutralizing titers in the sera of after NJ/76 vaccination was evaluated with the Spearman test for nonparametric correlation. r: Spearman r; P: two-tailed P value. The dotted lines in both panels A and B represent the neutralization titer of 160, which has been proposed as a correlate of seroprotection in microneutralization assays involving replicating influenza virus [4]. Protective titers based on neutralization of HA-pseudotypes have not been determined.

Because HA from A/New Jersey/1976 (NJ/76) and 2009 H1N1 influenza viruses are highly related (Figure 1), we first asked whether immunization in 1976 with the NJ/76 swine influenza vaccine could provide any immunity against the 2009 H1N1 influenza virus. Sixty five pre- and post-vaccination sera archived from the NJ/76 swine influenza vaccine trials conducted in 1976 [22] were evaluated for neutralizing activity to either NJ/76 or 2009 H1N1 A/Mexico/4108/2009 (Mex/4108/09) using HA-pseudotypes. Previously, we showed that HA-pseudotype neutralization titers using 95% inhibitory concentration (IC95) correlate well with conventional microneutralization titers using replicating influenza virus [23] and that HA-pseudotype neutralization is specific [21], [24]. Microneutralization titers >160 and a 4-fold increase after vaccination in assays involving replicating influenza virus have been proposed as correlates of seroprotection [4], but protective titers for HA-pseudotype neutralization have not yet been established. Positive control sera from 2009 H1N1 influenza virus infected ferrets typically have titers >10,000 [24]. Sera from the NJ/76 swine influenza vaccine trial were then tested and showed that NJ/76 vaccination generated neutralizing antibodies (titers >160 and a 4-fold increase after vaccination) in 85% and 77% of subjects against NJ/76 and Mex/4108/09 HA-pseudotypes, respectively (Table 1 and Figure 2A), consistent with the high degree of relatedness between the viruses and other recent reports [4], [8]. The neutralizing antibody titers to NJ/76 (GMT 597) and Mex/4108/09 (GMT 573) were also similar and correlated (Figure 2B). Most importantly, all sera with neutralization activity to NJ/76 showed significant neutralization activity to Mex/4108/09 (Figure 2B). Pre-vaccination sera did not exhibit significant neutralizing activity to HA-pseudotypes for either influenza virus, though titers against Mex/4108/09 (GMT 60) were higher than those against NJ/76 (GMT 3), suggesting that influenza viruses with shared epitopes to Mex/4108/09 influenza virus may have circulated previously.

**Table 1:** Summary of neutralization titers of sera from the NJ/76 swine influenza vaccine trials.

	Pre Vaccination		Post Vaccination	
Neutralization to	GMT (95% CI)	Percentage (titer >160)	GMT (95% CI)	Percentage (titer >160 and 4-fold increase)
NJ/76	3 (2–3)	0	597 (401–889)	85
Mex/4108/09	60 (45–81)	2	573 (399–825)	77
NCD/20/99	62 (44–88)	12	320 (246–417)	45
Bris/59/07	8 (6–11)	0	52 (38–70)	17

NJ/76: A/New Jersey/1976; Mex/4108/09: A/Mexico/4108/2009; NCD/20/99: A/New Caledonia/20/1999; Bris/59/07: A/Brisbane/59/2007.

**Figure 3:**
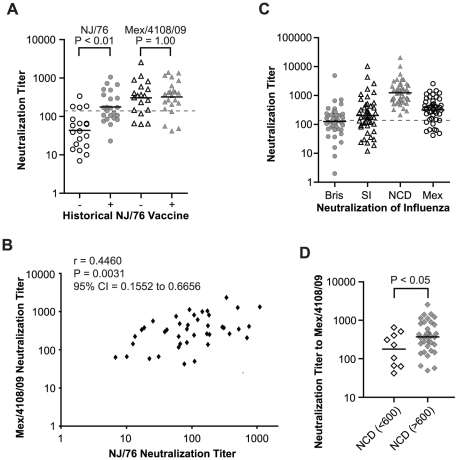
Relationship between 2009 H1N1 neutralization and immunizations with NCD/20/99 and NJ/76 vaccines. (A) Comparison of neutralization titers to NJ/76 and Mex/4108/09 in the contemporary cohort of subjects having received recent seasonal influenza vaccines with (+) and without (−) history of NJ/76 vaccination. P values were calculated by unpaired t test for the comparison of groups with (+) and without (−) history of NJ/76 vaccination. (B) Correlation between neutralizing titers to NJ/76 and Mex/4108/09 in the subjects aged 48–64 years with (+) and without (−) history of NJ/76 vaccination was evaluated with Spearman test for nonparametric correlation. r: Spearman r; P: two-tailed P value. (C) Neutralization titers to Bris/59/07, SI/03/06, NCD/20/99 and Mex/4108/09 in the subjects aged 48–64 years who had received all seasonal influenza vaccinations including Bris/59/07, SI/03/06 and NCD/20/99. (D) Neutralization titers to Mex/4108/09 in the subjects aged 48–64 years who had received all seasonal influenza vaccinations were stratified neutralization titer (>600) in NCD/20/99 vaccination. P values were calculated by unpaired t test for the comparison of the two stratified groups. The geometric mean of titers (GMT) of neutralization in each group is shown as a short dark line in panels A, C and D. The dotted lines in both panels A and C represent neutralization titer of 160, which has been proposed as a correlate of seroprotection in microneutralization assays involving replicating influenza virus [4]. Protective titers for neutralization of HA-pseudotypes have not been determined. Bris: Bris/59/07; SI: SI/03/06; NCD: NCD/20/99; Mex: Mex/4108/09.

We next asked whether subjects with a history of NJ/76 vaccination have significant neutralization titers to 2009 H1N1 today. Accordingly, we analyzed sera from a contemporary cohort of 23 subjects who had a history of NJ/76 vaccination and 19 aged-matched control subjects who did not. As shown in Table 2 and Figure 3A, sera from those who received the NJ/76 vaccine more than 30 years ago showed significant neutralization titers to NJ/76 (GMT 181), with 52% having neutralization titers >160. Sera from subjects who did not receive the NJ/76 vaccine had a GMT of only 44 to NJ/76, although a few individuals showed significant neutralization titers (>160). We note that the neutralization titers to NJ/76 in sera from subjects who did not receive the NJ/76 vaccine in this contemporary cohort were higher than the pre-vaccination sera in NJ/76 trials, suggesting that natural infection and/or vaccination with seasonal influenza strains during the period 1977–2009 provided a low level of cross-neutralization to NJ/76. Thus there appears to be residual immunity to NJ/76 in a majority of persons who were previously immunized with NJ/76 vaccine, or immunity may have been boosted by exposures during intervening years.

**Table 2:** Summary of neutralization titers of contemporary sera from subjects with or without a history of having received the NJ/76 swine influenza vaccine.

	No NJ/76 Vaccination		NJ/76 Vaccination	
Neutralization to	GMT (95% CI)	Percentage (titer >160)	GMT (95% CI)	Percentage (titer >160)
NJ/76	44 (26 – 74)	21	181 (121 – 270)	52
Mex/4108/09	305 (187 – 497)	68	331 (215 – 511)	83

NJ/76: A/New Jersey/1976; Mex/4108/09: A/Mexico/4108/2009.

We next assessed cross-neutralization to 2009 H1N1 HA-pseudotypes. Unexpectedly, titers among those immunized with the NJ/76 vaccine were higher against Mex/4108/09 (GMT 331) compared to NJ/76 (GMT 181), with 83% having neutralizing antibody titers ranging from 161–1456 (GMT 469). There was a significant correlation between neutralization titers to NJ/76 and Mex/4108/09 (Figure 3B). However, sera from subjects without a history of NJ/76 vaccination had similar cross-neutralization titers to Mex/4108/09 (GMT 305), with 68% having neutralization titers >160 (Table 2 and Figure 3A). The substantial neutralizing titers to Mex/4108/09 found in a high proportion of subjects in this contemporary cohort, regardless of their vaccination history to NJ/76, indicated that their cumulative history of influenza infections and vaccinations have involved strains that share neutralizing epitopes with the 2009 H1N1 influenza virus.

### Annual seasonal influenza vaccinations and cross-neutralizing antisera to 2009 H1N1

All 45 subjects in the contemporary cohort received all annual seasonal influenza vaccines for at least the past five years (2004/05–2008/09 seasons). To investigate potential correlations between neutralizing activity to recent seasonal H1N1 influenza and the 2009 H1N1 viruses, we tested all sera for HA-pseudotype neutralizing activity against the three recent seasonal H1N1 influenza strains, A/New Caledonia/20/1999 (NCD/20/99), A/Solomon Islands/3/2006 (SI/03/06), and A/Brisbane/59/2007 (Bris/59/07). NCD/20/99 was used 7 times in influenza vaccines during the 2000/01 to 2006/07 seasons. SI/03/06 and Bris/59/07 were used in the 2006/07 and 2008/09 seasonal influenza vaccines, respectively (www.fludb.org/brc/vaccineRecommend.do?decorator=influenza). Neutralization of HA-pseudotypes corresponding to each of these strains is specific, as shown in Table S1. Using these HA-pseudotypes, 100% of subjects showed significant neutralization titers against NCD/20/99 (GMT 1237) (Table 3 and Figure 3C), consistent with the repeated use of the NCD/20/99 strain in recent seasonal influenza vaccines. Only 49% and 60% had neutralization titers >160 against Bris/59/07 and SI/03/06, respectively (Table 3 and Figure 3C). By comparison, the GMT of neutralizing titers to 2009 H1N1 is 319, with 76% having neutralization titers >160, regardless of vaccination history to NJ/76 (Figure 3C). The neutralization titers to Mex/4108/09 did not correlate with the titers to Bris/59/07 and SI/03/06 (data not shown), but subjects with higher neutralization titers (>600) to NCD/20/99 showed higher cross-neutralization titers to Mex/4108/09 (p<0.05) (Figure 3D), suggesting that there may be shared neutralization epitopes between NCD/20/99 and Mex/4108/09.

**Table 3:** Summary of neutralization titers of contemporary sera from subjects with a history of having received seasonal influenza vaccines.

	Neutralization Titers	
Neutralization to	GMT (95% CI)	Percentage (titer >160)
Bris/59/07	127 (88 – 183)	49
SI/03/06	204 (133 – 315)	60
NCD/20/99	1237 (938 – 1631)	100
Mex/4108/09	319 (237 – 428)	76

Serum samples were from the subjects with Bris/59/07, SI/03/06 and NCD/20/99 vaccinations. Bris/59/07: A/Brisbane/59/2007; SI/03/06: A/Solomon Islands/3/2006; NCD/20/99: A/New Caledonia/20/1999; Mex/4108/09: A/Mexico/4108/2009.

### Cross-neutralizing antisera to 2009 H1N1 and NCD/20/99

To look for cross-neutralization between 2009 H1N1 and NCD/20/99, we analyzed sera collected in 1976 from the NJ/76 vaccine trials for neutralizing activity to NCD/20/99 HA-pseudotypes. Since persons participating in the NJ/76 swine influenza vaccine trial were presumably not previously exposed to NCD/20/99 through natural infection or by vaccination, we considered the presence of neutralizing activity to NCD/20/99 in these sera to be due to cross-neutralizing antibodies. We found that the post NJ/76 vaccination sera had significant cross-neutralization activity to NCD/20/99 (GMT 320) with 45% having neutralization titers >160 and a 4-fold increase over pre-immunization titers, while only 12% of the pre NJ/76 vaccination sera had significant neutralization titers (Table 1 and Figure 2A). The reason that several pre NJ/76 vaccination sera have significant neutralizing activity to NCD/20/99 may be due to prior infections with related viruses.

To determine whether NJ/76 vaccination elicits cross-neutralizing activity to other recent seasonal H1N1 viruses, we analyzed the sera for the presence of neutralizing antibodies to Bris/59/07. Neutralization of Bris/59/07 HA-pseudotypes was seen in only 17% sera with titers >160 and a 4-fold increase over pre-immunization titers. Although the titers to Bris/59/07 were low (GMT 52) after vaccination with NJ/76, they were significantly higher than the titers in the pre-vaccination group (GMT 8) (Table 1 and Figure 2A). However, NJ/76 vaccination elicited much less cross-neutralization to Bris/59/07 than to NCD/20/99.

### HA2 subunit involvement in cross-neutralization between 2009 H1N1 and NCD/20/99

The cross-neutralization activity seen in sera after immunization with NJ/76 and seasonal influenza vaccines suggested the presence of shared neutralization epitopes between 2009 H1N1 and NCD/20/99. Since neutralizing antibodies can target either HA1 or HA2, we next investigated which subunit of HA accounts for the majority of the cross-neutralization between 2009 H1N1 and NCD/20/99 observed in our sera.

**Figure 4:**
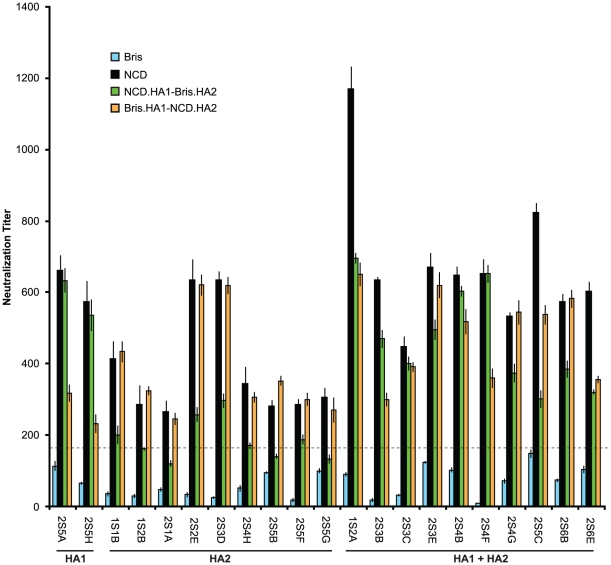
Cross-neutralization to NCD/20/99 from NJ/76 vaccination frequently maps to HA2. Bris.HA1-NCD.HA2 and NCD.HA1-Bris.HA2 pseudotypes were used to map the target of cross-neutralizing antibodies in the sera from the NJ/76 vaccine trials with neutralization titers to NCD/20/99, but not to Bris/59/07. The dotted line represents a neutralization titer of 160, which has been proposed as a correlate of seroprotection in microneutralization assays involving replicating influenza virus [4]. Protective titers for neutralization of HA-pseudotypes have not been determined. Data are shown as means +/− SD and reflect two or more independent experiments with each sample run in duplicate. Bris: Bris/59/07; NCD: NCD/20/99.

First we analyzed the sera from the NJ/76 vaccination trials. The sera with neutralization titers <160 to Bris/59/07 HA-pseudotypes were considered negative for neutralization to either HA1 or HA2 of Bris/59/07 HA. Twenty-one out of 65 post NJ/76 vaccination sera without neutralization activity to Bris/59/07, but with neutralization titers >160 and a 4-fold increase over pre-immunization titers to NCD/20/99 (neutralization titers <160 before vaccination), were identified (Table S2) and used for mapping. Chimeric HA involving the NCD/20/99 HA1 and Bris/59/07 HA2 subunits (NCD.HA1-Bris.HA2), as well the Bris/59/07 HA1 and NCD/20/99 HA2 subunits (Bris.HA1-NCD HA2) were constructed and used for making HA-pseudotypes. The infectivity and amount of HA in these chimeric HA-pseudotypes were comparable to the wild-type HA-pseudotypes (Figure S1). The chimeric HA-pseudotypes showed that: HA1 was responsible for most of the NCD/20/99 cross-neutralization in 2 out of 21 sera (e.g. 2S5H and 2S5A); HA2 was responsible for most of the NCD/20/99 cross-neutralization in 9 out of 21 sera (e.g. 2S5G, 2S5F, 2S5B, 2S4H, 2S3D, 2S2E, 2S1A, 1S2B and 1S1B); and both HA1 and HA2 were responsible for much of the NCD/20/99 cross-neutralization in 10 out of 21 sera (e.g. 2S6E, 2S6B, 2S5C, 2S4G, 2S4F, 2S4B, 2S3E, 2S3C, 2S3B and 1S2A) (Figure 4). In many cases, cross-neutralization titers to NCD/20/99 did not simply reflect the sum of the individual neutralization titers to each of the chimeras containing either NCD HA1 or HA2 subunits (e.g. 2S6B, 2S4G, 2S4F, 2S4B, 2S3E, 2S3D and 2S3C), indicating that HA1-HA2 interactions affected neutralization. These data suggested that there may be several targets for cross-neutralization. Nonetheless, the neutralization activity frequently mapped to the HA2 subunit, and in many cases, HA2 appeared to be the major determinant for cross-neutralization.

**Figure 5:**
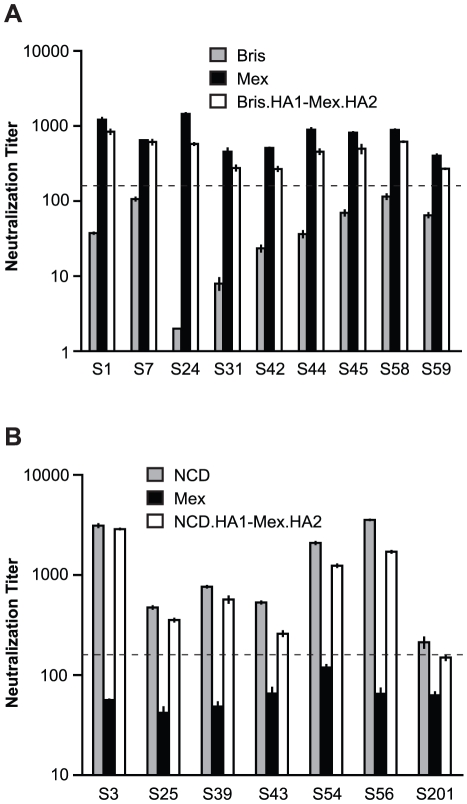
HA2 influences cross-neutralization between Mex/4108/09 and recent seasonal H1N1 influenza A strains. (A) Bris.HA1-Mex.HA2 pseudotypes were used to map the target of cross-neutralizing antibodies in the samples with cross-neutralization titers to Mex/4108/09, but not to Bris/59/07, in sera from the contemporary cohort of subjects who received seasonal influenza vaccines. (B) Neutralization to NCD/20/99, but not to Mex/4108/09, was mapped to NCD/20/99 HA1 using NCD.HA1-Mex.HA2 pseudotypes and sera from the contemporary cohort of subjects who received seasonal influenza vaccines. The dotted lines in both panels A and B represent the neutralization titer of 160, which has been proposed as a correlate of seroprotection in microneutralization assays involving replicating influenza virus [4]. Protective titers for neutralization of HA-pseudotypes have not been determined. Data are shown as means +/− SD and reflect two or more independent experiments with each sample run in duplicate. Bris: Bris/59/07; Mex: Mex/4108/09; NCD: NCD/20/99.

Next we analyzed the sera from the contemporary cohort. Sera with cross-neutralization titers (>160) to Mex/4108/09, but without neutralization titers (<160) to Bris/59/07 were identified (Table S3) and used for evaluating neutralizing antibodies that may be directed to Mex/4108/09 HA1 and/or HA2 subunits. HA-pseudotypes carrying the chimeric HA consisting of Bris/59/07 HA1 and Mex/4108/09 HA2 (Bris.HA1-Mex.HA2) showed that neutralization titers to Mex/4108/09 HA and Bris.HA1-Mex.HA2 were similar in all comparisons (samples S1, S7, S24, S31, S42, S44, S45, S58 and S59) (Figure 5A), suggesting that cross-neutralization to Mex/4108/09 involves the Mex/4108/09 HA2 subunit. Curiously, the chimeric Mex.HA1-Bris.HA2 HA-pseudotypes did not have high enough infectivity for neutralization studies, despite good HA incorporation and cleavage of HA0 in the HA-pseudotypes (Figure S1). Therefore, we could not directly assess the contributions of the Mex/4108/09 HA1 subunit to cross-neutralization.

To confirm the reliability of the cross-neutralizing data involving chimeric HA-pseudotypes with the Mex/4108/09 HA2 subunit, we identified sera with neutralization titers (>160) to NCD/20/99, but without cross-neutralization titers (<160) to Mex/4108/09 (Table S4). For these sera (samples S3, S25, S39, S43, S54, S56 and S201), neutralization titers for HA-pseudotypes carrying the chimeric NCD/20/99 HA1 and Mex/4108/09 HA2 (NCD.HA1-Mex.HA2) or NCD/20/99 HA were similar (Figure 5B), indicating that neutralization antibodies were directed to the NCD/20/99 HA1 subunit. Therefore, the presence of the Mex/4108/09 HA2 subunit in chimeric HA does not apparently give spurious neutralization results. Again, we were unable to assess neutralization of the complementary chimeric HA-pseudotypes containing Mex/4108/09 HA1 (Mex.HA1-NCD.HA2) due to the poor infectivity of this chimera, despite good incorporation of mature chimeric HA into the HA-pseudotypes (Figure S1). The difficulties in generating functional chimeric HA involving Mex/4108/09 HA1 further suggests that there are interactions between the Mex/4108/09 HA1 and HA2 subunits that are not present in recent seasonal H1N1 HA.

### Epitopes of cross-neutralization in HA2

**Figure 6:**
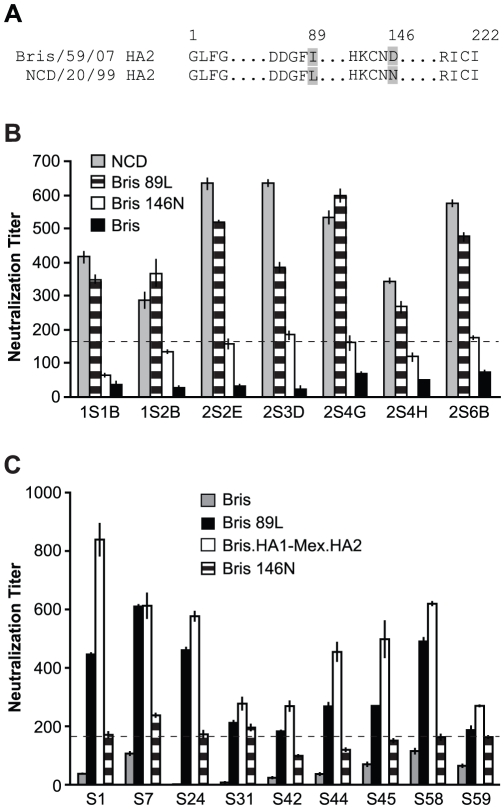
The amino acid at position 89 in HA2 influences neutralization to HA2. (A) Sequence comparison of HA2 from Bris/59/07 and NCD/20/99. The differing residues in the HA2 are indicated (grey). (B) The 89L mutation, but not the 146N mutation in HA2 of Bris/59/07, conferred similar levels of neutralization to Bris/59/07 HA-pseudotypes as compared to NCD/20/99 HA-pseudotypes, using sera from the NJ/76 vaccine trials that have neutralization titers to NCD/20/99, but not to wild-type Bris/59/07. (C) The 89L mutation, but not the 146N mutation in Bris/59/07 HA2, conferred similar levels of neutralization to Bris/59/07 HA-pseudotypes as compared to chimeric HA-pseudotypes with Mex/4108/09 HA2, using the contemporary sera cohort from samples that have neutralization titers to Mex/4108/09 HA2 (determined in Figure 5A), but not to wild type Bris/59/07. The dotted lines in both panels B and C represent the neutralization titer of 160, which has been proposed as a correlate of seroprotection in microneutralization assays involving replicating influenza virus [4]. Protective titers for neutralization of HA-pseudotypes have not been determined. Data are shown as means +/− SD and reflect two or more independent experiments with each sample run in duplicate. Bris: Bris/59/07; Mex: Mex/4108/09; NCD: NCD/20/99.

In 1993 [14] and again in a number of recent studies [15]–[20], neutralizing monoclonal antibodies that are broadly active against many influenza subtypes have been identified and mapped to epitopes in the stalk regions of the HA2 subunit [14], [16]–[20]. Although some of the cross-neutralization that we observed in our sera appears to map to the HA2 subunit, our data indicated that this cross-neutralization may be strain specific. As shown in Figure 4, Figure 5A and Figure 6, we found that sera with cross-neutralization to NCD/20/99 and Mex/4108/09 HA2 did not neutralize Bris/59/07. Significantly, there are only two amino acid differences in HA2, at the positions 89 (415 in full HA) and 146 (472 in full HA) between NCD/20/99 and Bris/59/07 HA2 (Figure 6A), suggesting that these two amino acids could influence HA2 antigenicity. When a leucine at residue 89 in HA2 (89L) or an asparagine at position 146 in HA2 (146N) corresponding to NCD/20/99 HA2 were introduced into Bris/59/07 HA2, the sera without cross-neutralization to Bris/59/07 HA showed neutralization to Bris/59/07 HA2-89L, but not to Bris/59/07 HA2-146N, with titers similar to NCD/20/99 HA and Bris.HA1-Mex.HA2 (Figure 6B and 6C). When both 89L and 146N were presented in Bris/59/07 HA2, serum titers were the same as those to Bris.HA1-NCD.HA2 in Figure 4 (data not shown). These results demonstrated that the neutralization epitopes in HA2 were influenced by residue 89 in HA2 (415 in full HA).

We then reviewed human H1N1 influenza virus HA sequences (www.fludb.org/brc/home.do?decorator=influenza) and noted that leucine at position 89 in HA2 has been maintained in seasonal H1N1 influenza viruses from at least 1918 to 2005 (Table 4). During this period, there are only two exceptions: A/Denver/1/1957 from North America has a methionine and A/Canterbury/106/2004 from Oceania has an isoleucine at position 89 of HA2. The change of leucine to isoleucine at position 89 of HA2 appeared frequently in 2006 with about 37.8% strains containing isoleucine, and the change of leucine to isoleucine continued in 2007 with about 34.1% strains containing isoleucine. However, by 2008, isoleucine completely replaced leucine at position 89 in HA2, raising the possibility that this change may reflect immune escape.

**Table 4:** Evolutionary changes of residue 89 in human H1N1 HA2.

Year	Residue 89 in HA2 (percentage)
1918–2005	L (100%)*
2006	L (62.2%) and I (37.8%)
2007	L (65.9%) and I (34.1%)
2008	I (100%)

Human H1N1 HA sequences with residue 89 in HA2 in database (www.fludb.org/brc/home.do?decorator=influenza) were compared for the evolutionary changes of residue 89.

*: Except M89 in A/Denver/1/1957 HA2, and I89 in A/Canterbury/106/2004 HA2.

## Discussion

The 2009 H1N1 HA diverges considerably from recent seasonal H1N1 HA and is more closely related to the NJ/76 HA (Figure 1), raising doubts about the extent of protection that could be afforded by vaccination with recent seasonal influenza vaccines. Our studies show that sera from the NJ/76 swine influenza vaccine trials and contemporary sera from subjects who received recent seasonal influenza vaccines, regardless of whether they had been immunized with the NJ/76 swine influenza vaccine, frequently have cross-neutralizing activity to the 2009 H1N1. Further, these sera revealed one or more cross-neutralization epitopes that were sensitive to a conservative amino acid change in position 89 in the HA2 subunit, corresponding to a naturally-occurring amino acid variant that emerged in seasonal H1N1 influenza viruses in recent years.

Several groups have reported that prior infections or vaccinations can confer some immunity to 2009 H1N1, though findings vary. There is agreement that individuals >65 years have substantial cross-reactive antibodies to the 2009 H1N1, consistent with the epidemiology of the 2009 H1N1 pandemic showing that younger age groups were disproportionately affected [4], but the extent of cross-immunity induced by recent seasonal influenza vaccines is more ambiguous [4]–[9], [25]–[29]. Differences in methodologies and history of vaccination or infection with NCD/20/99 may have affected the outcomes. Our results involving persons aged 48–64 years (Table S5) extend other reports showing that older persons generally have some pre-existing immunity to the 2009 H1N1, but more significantly highlight the presence of cross-neutralizing antibodies between 2009 H1N1 and NCD/20/99. Because all subjects in our contemporary cohort received yearly seasonal influenza vaccines for at least the past five years, and NCD/20/99 was repeatedly used in seasonal vaccines during the 2000/01–2006/07 influenza seasons, we cannot determine the extent to which influenza vaccinations and/or natural infections contributed to the generation of cross-neutralizing antibodies to 2009 H1N1 and NCD/20/99.

To investigate potential cross-neutralizing determinants in NCD/20/99 and 2009 H1N1, we used chimeric HA-pseudotypes involving HA1 and HA2 subunits of NCD/20/99 and Bris/59/07 and sera that lacked neutralization to Bris/59/07 (Table S2). Both contemporary and archived sera from the NJ/76 swine influenza vaccine trials contained cross-neutralizing antibodies that depended on the HA2 subunit (Figure 4 and 5). Most remarkable, we found that the cross-neutralization was influenced by a single conservative amino acid change at position 89 in HA2, which differed between NCD/20/99 and Bris/59/07 (Figure 6). Thus, these data reveal a new determinant in the C helix region of the HA2 stalk that modified sensitivity to cross-neutralizing antibodies present in human sera from two different cohorts separated by more than three decades.

**Figure 7:**
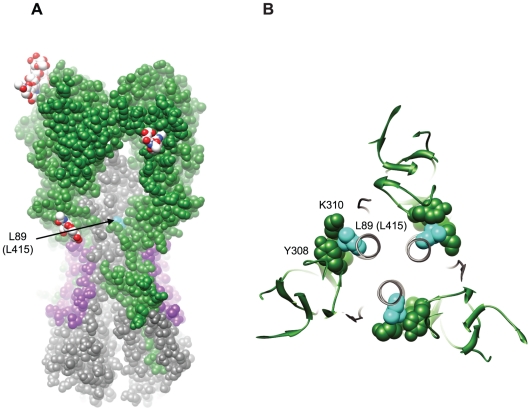
Structural implications for the L89I (L415I) mutation in HA2. (A) Space filling representation of the surface of A/California/04/2009 HA (PDB:3LZG) to show the recessed location of L89 (L415) (cyan). HA1 is colored green; HA2 is colored gray; the HA2 stem epitope structurally defined in Sui *et al.* [18] and Ekiert *et al.* [17] is colored purple; and glycans at glycosylation sites are colored by heteroatom (carbon white, oxygen red, and nitrogen blue). (B) Depiction of the packing interaction between L89 (L415) and residues in an adjacent loop in HA1 (PDB:3LZG). Molecular graphics images were produced using the UCSF Chimera package (supported by NIH P41 RR001081) [45].

Growing interest in the generation of broadly neutralizing influenza antibodies has led to the discovery of several new monoclonal antibodies that bind to HA2 [14]–[20], [30], [31]. The first reported heterosubtypic neutralizing antibody, C179, derived from a mouse immunized with the A/Okuda/57 H2N2 strain, was found to be directed to a conformational epitope involving the A helix in the HA2 stalk (Figure 7A) and a region in HA1 [14]. More recently, several other HA2 heterosubtypic neutralizing monoclonal antibodies that are potent against strains from H1 and H5 subtype (Group 1) influenza viruses have been isolated using various methods. Some of these antibodies have been also shown to make contacts with the A helix of HA2 [16], [17], [30] (Figure 7A). Other HA2 monoclonal antibodies have been shown to bind to a highly conserved pocket in the stalk region containing the fusion peptide [18] or undetermined regions of the HA2 stalk [19]. Another potent broadly neutralizing monoclonal antibody against H3N2 (Group 2) but not H1N1 (Group 1) strains was shown to bind to a peptide corresponding to the C helix region in the HA2 stalk [20].

The HA2 monoclonal antibodies bind to regions in the HA2 stalk and interfere with conformational changes that are needed for virus entry [32], but they do not block HA attachment to receptors. These HA2 antibodies lack HI activity and were discovered using neutralization assays that sometimes involved HA-pseudotypes [18]–[20]. We [21], [23] and others [33], [34] have shown that HA-pseudotypes neutralization titers are highly correlated with microneutralization titers for replicating influenza virus, but the correlate of protection using HA-pseudotype neutralization titers has not been determined. Also, glycoproteins on the surface of HIV-based retroviral particle may be less densely packed and more exposed compared to HA on the surface of influenza viral particles, perhaps making them more susceptible to HA2-directed neutralization compared to influenza virus, as suggested in some studies [18], [19]. While sensitive screening assays have allowed many groups to fish out broadly neutralizing antibodies, it is generally believed that HA2 heterosubtypic neutralizing antibodies are present at relatively low concentrations, as compared with antibodies directed to HA1 [19], [35]. The need to change annual seasonal influenza vaccines to match dominant circulating strains indicates that such HA2 cross-neutralizing antibodies may not be present at high enough titers to provide robust protection. It is therefore difficult to discern the degree to which HA2 antibodies in our sera samples could contribute to protection to 2009 H1N1 virus. However, studies in animal models have provided proof of concept that induction [16], [18]–[20] or passive transfer of HA2 antibodies alone [20], [36] can provide protection. Appropriately designed vaccines may be able to induce robust immune responses to conserved neutralizing epitopes in HA2 [35], [37]. Recent examples involving several approaches are showing promise [20], [38], [39].

Our finding that the conservative substitution of isoleucine for 89 L reduced sensitivity to cross-neutralizing antibodies present in our sera was surprising. The crystal structure of the A/Cal/04/2009 HA [40] shows that 89 L packs tightly into a poorly exposed crevice underneath the HA1 crown (Figure 7A), making intimate contact with HA1 through a lysine and tyrosine at residues 310 and 308, respectively (Figure 7B). Substitution of 89 L with isoleucine may cause the interactions between HA1 and HA2 in this region to shift in order to accommodate the alternate side chain (Figure S2), and in doing so, could directly alter exposure or conformation of the antibody binding site. Alternatively, residue 89 may be evolving in response to immune pressure at distant sites. For example, 89I may reflect an adaptive change in HA2 resulting from direct immune pressure on epitopes in HA1. The 89I substitution could also impose allosteric changes on nearby or more distant neutralizing epitopes in either HA1 or HA2. The observation that Bris/59/07 was less sensitive to neutralization by an HA2 antibody compared to NCD/20/99 is consistent with the notion that this residue could influence neutralization by HA2 antibodies [19]. We also note that 89L is not near any of the contact residues for the recently described HA2 monoclonals specific for Group 1 HAs, although it is located on the C helix region of the HA2 stalk that has recently been suggested to contain an epitope for the 12D1 monoclonal antibody that binds H3 strains from Group 2.

Review of the database of human H1N1 HA also offers intriguing clues about the potential significance of the change of leucine to isoleucine at position 89 in HA2. We note that 89L has been maintained in seasonal H1N1 influenza viruses from at least 1918 until 2006 when it started to change to isoleucine, and 89L disappeared in 2008 (Table 4). It is tempting to speculate that this change could reflect immune escape. We also note that H3 strains from Group 2 influenza viruses generally have an isoleucine at the corresponding position in HA2. Interestingly, unlike Group 1 H1N1 HA, a carbohydrate can be seen in the H3N2 HA crystal structure extending in the vicinity of the isoleucine (coming from N285) (PDB 3HMG) [41], [42], which could conceivably have evolved to shield it from neutralizing antibodies. These observations offer a cautionary note that antigenic drift in this region may arise under strong selection pressure. Nonetheless, the viable substitutions may be limited due to the fact that residue 89 and others in the stalk regions make important contacts in both the native and low pH structures of HA, consistent with the difficulties in generating escape mutants with some of the HA monoclonal antibodies [18], [20]. Perhaps this explains why H3N2 strains have incorporated a carbohydrate in the vicinity of this region.

In summary, our studies showed that cross-neutralizing antibodies to 2009 H1N1 influenza that involve the HA2 subunit could be detected in sera collected in 1976 from NJ/76 swine influenza vaccine trials and sera from persons aged 48–64 who received annual influenza vaccines for at least the past five years. A conservative substitution at position 89 in HA2, found in drifted seasonal influenza virus variants from the 2006/07 and 2007/08 influenza seasons, abrogated this neutralization. Future studies involving vaccines that elicit strong antibody responses to HA2 will reveal the extent to which mutations can lead to immune escape.

## Materials and Methods

### Viruses, plasmids, and cell lines

Full-length HA ORF with Q223R mutation from A/Mexico/4108/2009 (GenBank GQ223112) and full-length wild type HA ORFs from A/Solomon Islands/3/2006 (GenBank EU100724), A/New Caledonia/20/1999 (GenBank AY289929), and A/Brisbane/59/2007 (GenBank CY058487) were amplified from viruses by reverse transcription-polymerase chain reaction (RT-PCR). Full-length wild type NA ORF from A/California/04/2009 (GenBank FJ966084) was also amplified from virus by RT-PCR. Full-length wild type HA ORF of A/New Jersey/1976 (GenBank CY021957) was chemically synthesized by GenScript (Piscataway, NJ). Chimeric HA carrying HA1 and HA2 from different strains were constructed by ligation of PCR fragments of HA1 and HA2. The HA and NA ORFs were then placed into the pCMV/R expression plasmid obtained from Dr. Gary J. Nabel (National Institutes of Health (NIH), Bethesda, MD), as described previously [21]. Full-length wild type M2 ORF of A/Puerto Rico/8/1934 (GenBank EF467824) was chemically synthesized by Integrated DNA Technologies (Coralville, IA) and placed into pCDNA 3.1(+) (Invitrogen, Carlsbad, CA). Codon-optimized human airway trypsin-like protease (HAT) gene expression construct (pCAGGS-HATcop) was described before [23]. The HIV gag/pol (pCMVΔR8.2) and Luc reporter (pHR'CMV-Luc) constructs were described previously [43], [44] and obtained from Dr. Gary J. Nabel (NIH, Bethesda, MD).

293T cells were cultured in Dulbecco's modified eagle medium (DMEM) with high glucose, L-Glutamine, MEM non-essential amino acids, penicillin/streptomycin and 10% fetal calf serum.

### Ethics statement

Ethics approval by the Research Involving Human Subjects Committee (RIHSC) at the US Food and Drug Administration was obtained for use of the sera involved in this study. Under 45 CFR 46.101 (b) (4), the sera from the 1976 swine influenza trial was included in the category of exempt research because the study used only existing sera, and information was recorded in such a manner that subjects can not be identified, either directly or through identifiers (RIHSC Protocol #09-043B). The sera from the contemporary cohort were obtained with written informed consent from all participants (RIHSC Protocol #09-110B).

### Serum samples

Two groups of human sera were used in this study. The sera in group one included frozen samples retrieved from storage at FDA/CBER involving 65 pre-vaccination and post-vaccination sera from A/New Jersey/1976 swine influenza vaccine trials conducted in 1976 [22]. The sera in group two were collected in September-December of 2009 from 45 volunteers aged 48–64 years, without a history of vaccinations or influenza symptoms or exposures in 2009. All subjects in group two received at least five year (2004/05 to 2008/09) annual seasonal influenza vaccines including A/New Caledonia/20/1999, A/Solomon Islands/3/2006 and A/Brisbane/59/2007 used for the seasons from 2000/01 to 2008/09, and 23 subjects among them also received the A/New Jersey/1976 swine influenza vaccine (Table S5). Sera were heat inactivated by incubation at 56°C for 30 minutes prior to use in neutralization assays. Sera were assessed for neutralizing antibodies to 2009 H1N1 (A/Mexico/4108/2009) and the 2000/09 seasonal H1N1 influenza viruses (A/New Caledonia/20/1999, A/Solomon Islands/3/2006, A/Brisbane/59/2007) using an HA-pseudotype neutralization assay, as described below.

### Production of HA-pseudotypes

HA-pseudotypes carrying a luciferase (Luc) reporter gene were produced in 293T cells as described previously [21]. 2.5 µg of HAT, 2.5 µg of A/Puerto Rico/8/1934 M2, and 4 µg of A/California/04/2009 NA expression plasmids were included in the transfection. HA-pseudotypes were collected 48 hr post-transfection, filtered through a 0.45-µm low protein binding filter, and used immediately or stored at −80°C. HA-pseudotype titers were measured by infecting 293T cells with HA-pseudotypes for 48 hr prior to measuring luciferase activity in infected cells (luciferase assay reagent, Promega) as described previously [21]. HA-pseudotype titers were expressed as relative luminescence unit per milliliter of HA-pseudotype supernatants (RLU/ml).

### HA-pseudotype neutralization assay

As previously described [23], [24], HA-pseudotypes containing approximately 15 ng/ml of p24 antigen and 12 ng/ml of HA were incubated with heat-inactivated serum samples for 1 hr at 37°C, then 100 µl of HA-pseudotypes and serum mixtures were inoculated onto 96-well plates that were seeded with 2 x 10^4^ 293T cells/well one day prior to infection. HA-pseudotype infectivity was evaluated 48 hr later by luciferase assay, as previously described [21]. The serum dilution causing a 95% reduction of RLU compared to control (IC95-neutralizing antibody titer) was used as the neutralization endpoint titer [23]. IC95 was calculated using Graphpad Prism software. Data reported were from at least two independent experiments, with each serum sample run in duplicate.

### Statistical analysis

To evaluate vaccination responses and potential cross-protection, sera with neutralization titers over 160 that inhibited 95% infectivity were considered highly significant [4], [23]. The neutralization titers were analyzed with nonlinear regression using GraphPad Prism software. The correlation of neutralization titers was evaluated with Spearman's p, a test for nonparametric correlation. t-test, geometric mean titer (GMT) with 95% confidence intervals and corresponding P value were analyzed using GraphPad Prism software. P values <0.05 were considered statistically significant.

## Supporting Information

Figure S1 HA incorporation and Infectivity of chimeric HA-pseudotypes. (A) HA content in chimeric and wild-type HA-pseudotypes were similar. HA protein in HA-pseudotypes containing 10 ng of p24 antigen was detected by Western blot using rabbit H1 HA1 antiserum. (B) Infectivity of chimeric and wild-type HA-pseudotypes was similar for Bris/59/07 and NCD/20/99 strains, but chimeras involving HA1 of Mex/4108/09 were impaired. Infectivity is shown as the mean +/− S.D. of three HA-pseudotype stocks run in triplicate. Bris: Bris/59/07; NCD: NCD/20/99; Mex: Mex/4108/09.
(EPS)

Figure S2 #Side chain packing differences at residue 89 in H1N1 and H3N2 HA. H1N1 HA (PDB:3LZG) (A) and H3N2 HA (PDB:3HMG) (B) structures were aligned using the H1N1 HA2 as a reference in the visualization program Chimera [45]. In both panels residues from HA1 are green and HA2 are gray. Position 89 is colored cyan. Side chain interactions between position 89 and adjacent residues (red dashed lines) were then detected using UCSF's Chimera structural analysis tools (http://www.cgl.ucsf.edu/chimera). Despite the overall structural similarity in this region, the change in side chain branching between leucine and isoleucine permits a different pattern of van der Waals contacts between position 89 and the surrounding area. Red arrow indicates directionality of side chain contacts. Some of the residues contacting position 89 are not conserved between H1N1 and H3N2 HAs. Most notably, position 299 is a proline in H1N1 and a lysine in H3N2. The shift in packing, although subtle, illustrates that even a conservative leucine to isoleucine mutation in the context of an H1N1 HA has the possibility to create a packing mismatch that might alter the local structure and/or dynamics, which through its interface with HA1, may consequently be transmitted elsewhere in HA.
(EPS)

Table S1 Comparison of neutralization titers for H1N1 HA-pseudotypes. The Mex/4108/09, NCD/20/99, Bris/59/07 and SI/03/06 HA-pseudotypes were evaluated for neutralization by reference antisera. The 95% neutralization (IC95) titers represent at least duplicate testing. HA-pseudotypes: Mex/4108/09: A/Mexico/4108/2009; NCD/20/99: A/New Caledonia/20/1999; Bris/59/07: A/Brisbane/59/2007; SI/03/06: A/Solomon Islands/03/2006. Ferret antiserum: Cal/07/09: A/California/07/2009 (ATCC); NCD/20/99: A/New Caledonia/20/1999 (F-99-4A, FDA); Bris/59/07: A/Brisbane/59/2007 (2008-587, FDA); SI/03/06: A/Solomon Islands/03/2006 (2007-150, FDA).
(DOC)

Table S2 Summary of NJ/76 vaccination trial samples with neutralization titers to NCD/20/99 (>160, and 4-fold increase), Bris/59/07 (<160), and Mex/4108/09. NJ/76: A/New Jersey/1976; NCD/20/99: A/New Caledonia/20/1999; Bris/59/07: A/Brisbane/59/2007; Mex/4108/09: A/Mexico/4108/2009.
(DOC)

Table S3 Summary of seasonal influenza vaccination samples with neutralization titers to Mex/4108/09 (>160) and Bris/59/07 (<160). Mex/4108/09: A/Mexico/4108/2009; Bris/59/07: A/Brisbane/59/2007.
(DOC)

Table S4 Summary of seasonal influenza vaccination samples with neutralization titers to NCD/20/99 (>160) and Mex/4108/09 (<160). Mex/4108/09: A/Mexico/4108/2009; NCD/20/99: A/New Caledonia/20/1999.
(DOC)

Table S5 Summary of demographic information of subjects who received seasonal influenza vaccines. Samples S22, S27 and S37 are not included in the comparison of NJ/76 and Mex/4108/09 in Table 2, Figure 3A–B.
(DOC)
