# Supplementary material for: Cross-Neutralizing Antibodies to Pandemic 2009 H1N1 and Recent Seasonal H1N1 Influenza A Strains Influenced by a Mutation in Hemagglutinin Subunit 2
Source: PLoS Pathog. 2011 Jun 9;7(6):e1002081. doi: 10.1371/journal.ppat.1002081 (PMC3111540; doi:10.1371/journal.ppat.1002081)
Supplement: Table S1 — Comparison of neutralization titers for H1N1 HA-pseudotypes. The Mex/4108/09, NCD/20/99, Bris/59/07 and SI/03/06 HA-pseudotypes were evaluated for neutralization by reference antisera. The 95% neutralization (IC95) titers represent at least duplicate testing. HA-pseudotypes: Mex/4108/09: A/Mexico/4108/2009; NCD/20/99: A/New Caledonia/20/1999; Bris/59/07: A/Brisbane/59/2007; SI/03/06: A/Solomon Islands/03/2006. Ferret antiserum: Cal/07/09: A/California/07/2009 (ATCC); NCD/20/99: A/New Caledonia/20/1999 (F-99-4A, FDA); Bris/59/07: A/Brisbane/59/2007 (2008-587, FDA); SI/03/06: A/Solomon Islands/03/2006 (2007-150, FDA). (DOC) [file ppat.1002081.s003.doc]

**Table S1**

Comparison of neutralization titers for H1N1 HA-pseudotypes. The Mex/4108/09, NCD/20/99, Bris/59/07 and SI/03/06 HA-pseudotypes were evaluated for neutralization by reference antisera. The 95% neutralization (IC95) titers represent at least duplicate testing.

|  | Ferret antiserum | | | |
| --- | --- | --- | --- | --- |
| HA-pseudotypes | Cal/07/09 | NCD/20/99 | Bris/59/07 | SI/03/06 |
| Mex/4108/09 | 16922 | <80 | <80 | <80 |
| NCD/20/99 | <80 | 16757 | 552 | 1502 |
| Bris/59/07 | <80 | 1270 | 12432 | 4647 |
| SI/03/06 | <80 | 638 | 2265 | 28428 |

HA-pseudotypes:

Mex/4108/09: A/Mexico/4108/2009

NCD/20/99: A/New Caledonia/20/1999

Bris/59/07: A/Brisbane/59/2007

SI/03/06: A/Solomon Islands/03/2006

Ferret antiserum:

Cal/07/09: A/California/07/2009 (ATCC)

NCD/20/99: A/New Caledonia/20/1999 (F-99-4A, FDA)

Bris/59/07: A/Brisbane/59/2007 (2008-587, FDA)

SI/03/06: A/Solomon Islands/03/2006 (2007-150, FDA)
