# Supplementary material for: Cross-Neutralizing Antibodies to Pandemic 2009 H1N1 and Recent Seasonal H1N1 Influenza A Strains Influenced by a Mutation in Hemagglutinin Subunit 2
Source: PLoS Pathog. 2011 Jun 9;7(6):e1002081. doi: 10.1371/journal.ppat.1002081 (PMC3111540; doi:10.1371/journal.ppat.1002081)
Supplement: Table S2 — Summary of NJ/76 vaccination trial samples with neutralization titers to NCD/20/99 (>160, and 4-fold increase), Bris/59/07 (<160), and Mex/4108/09. NJ/76: A/New Jersey/1976; NCD/20/99: A/New Caledonia/20/1999; Bris/59/07: A/Brisbane/59/2007; Mex/4108/09: A/Mexico/4108/2009. (DOC) [file ppat.1002081.s004.doc]

**Table S2**

Summary of NJ/76 vaccination trial samples with neutralization titers to NCD/20/99 (>160, and 4-fold increase), Bris/59/07 (<160), and Mex/4108/09

|  | Neutralization Titers | | | | |
| --- | --- | --- | --- | --- | --- |
|  | Against NCD/20/99 | | | Against Bris/59/07 | Against Mex/4108/09 |
| Samples | Pre Vaccination | Post Vaccination | Titer Change (Fold) | Post Vaccination | Post Vaccination |
| 1S1B | 46 | 415 | 9 | 35 | 515 |
| 1S2A | 67 | 1171 | 17 | 90 | 649 |
| 1S2B | 5 | 286 | 57 | 29 | 163 |
| 2S1A | 17 | 265 | 16 | 47 | 1099 |
| 2S2E | 19 | 634 | 33 | 33 | 604 |
| 2S3B | 159 | 637 | 4 | 18 | 413 |
| 2S3C | 80 | 449 | 6 | 32 | 588 |
| 2S3D | 68 | 635 | 9 | 25 | 1058 |
| 2S3E | 15 | 671 | 45 | 123 | 1174 |
| 2S4B | 133 | 648 | 5 | 102 | 441 |
| 2S4F | 147 | 652 | 4 | 9 | 722 |
| 2S4G | 104 | 533 | 5 | 71 | 506 |
| 2S4H | 41 | 345 | 8 | 51 | 1158 |
| 2S5A | 149 | 661 | 4 | 113 | 1579 |
| 2S5B | 54 | 282 | 5 | 94 | 463 |
| 2S5C | 157 | 823 | 5 | 148 | 589 |
| 2S5F | 56 | 286 | 5 | 18 | 947 |
| 2S5G | 1 | 307 | 307 | 100 | 687 |
| 2S5H | 136 | 574 | 4 | 66 | 346 |
| 2S6B | 58 | 535 | 9 | 74 | 435 |
| 2S6E | 37 | 604 | 16 | 103 | 1397 |

NJ/76: A/New Jersey/1976; NCD/20/99: A/New Caledonia/20/1999; Bris/59/07: A/Brisbane/59/2007; Mex/4108/09: A/Mexico/4108/2009
