# Supplementary material for: Cross-Neutralizing Antibodies to Pandemic 2009 H1N1 and Recent Seasonal H1N1 Influenza A Strains Influenced by a Mutation in Hemagglutinin Subunit 2
Source: PLoS Pathog. 2011 Jun 9;7(6):e1002081. doi: 10.1371/journal.ppat.1002081 (PMC3111540; doi:10.1371/journal.ppat.1002081)
Supplement: Table S3 — Summary of seasonal influenza vaccination samples with neutralization titers to Mex/4108/09 (>160) and Bris/59/07 (<160). Mex/4108/09: A/Mexico/4108/2009; Bris/59/07: A/Brisbane/59/2007. (DOC) [file ppat.1002081.s005.doc]

**Table S3**

Summary of seasonal vaccination samples with neutralization titers to Mex/4108/09 (>160) and Bris/59/07 (<160)

|  | Neutralization Titers | |
| --- | --- | --- |
| Samples | Against Mex/4108/09 | Against Bris/59/07 |
| S1 | 1213 | 38 |
| S7 | 646 | 107 |
| S24 | 1456 | 2 |
| S31 | 456 | 8 |
| S42 | 512 | 24 |
| S44 | 897 | 37 |
| S45 | 816 | 70 |
| S58 | 886 | 115 |
| S59 | 402 | 65 |

Mex/4108/09: A/Mexico/4108/2009; Bris/59/07: A/Brisbane/59/2007
