# Supplementary material for: Cross-Neutralizing Antibodies to Pandemic 2009 H1N1 and Recent Seasonal H1N1 Influenza A Strains Influenced by a Mutation in Hemagglutinin Subunit 2
Source: PLoS Pathog. 2011 Jun 9;7(6):e1002081. doi: 10.1371/journal.ppat.1002081 (PMC3111540; doi:10.1371/journal.ppat.1002081)
Supplement: Table S4 — Summary of seasonal influenza vaccination samples with neutralization titers to NCD/20/99 (>160) and Mex/4108/09 (<160). Mex/4108/09: A/Mexico/4108/2009; NCD/20/99: A/New Caledonia/20/1999. (DOC) [file ppat.1002081.s006.doc]

**Table S4**

Summary of seasonal vaccination samples with neutralization titers to NCD/20/99 (>160) and Mex/4108/09 (<160)

|  | Neutralization Titers | |
| --- | --- | --- |
| Samples | Against Mex/4108/09 | Against NCD/20/99 |
| S3 | 57 | 3119 |
| S25 | 42 | 474 |
| S39 | 49 | 766 |
| S43 | 66 | 533 |
| S54 | 119 | 2093 |
| S56 | 65 | 3558 |
| S201 | 63 | 213 |

Mex/4108/09: A/Mexico/4108/2009; NCD/20/99: A/New Caledonia/20/1999
