# Supplementary material for: Cross-Neutralizing Antibodies to Pandemic 2009 H1N1 and Recent Seasonal H1N1 Influenza A Strains Influenced by a Mutation in Hemagglutinin Subunit 2
Source: PLoS Pathog. 2011 Jun 9;7(6):e1002081. doi: 10.1371/journal.ppat.1002081 (PMC3111540; doi:10.1371/journal.ppat.1002081)
Supplement: Table S5 — Summary of demographic information of subjects who received seasonal influenza vaccines. Samples S22, S27 and S37 are not included in the comparison of NJ/76 and Mex/4108/09 in Table 2, Figure 3A–B. (DOC) [file ppat.1002081.s007.doc]

**Table S5** Summary of demographic information of seasonal vaccination subjects

| Patient ID | Age | Sex | 1976 immunization | Date of bleed |
| --- | --- | --- | --- | --- |
| S5 | 59 | F | No | 10/14/2009 |
| S9 | 51 | F | No | 9/10/2009 |
| S12 | 58 | F | No | 12/11/2009 |
| S14 | 53 | M | No | 12/11/2009 |
| S42 | 51 | F | No | 10/15/2009 |
| S43 | 53 | F | No | 10/15/2009 |
| S44 | 62 | F | No | 10/15/2009 |
| S45 | 51 | M | No | 10/15/2009 |
| S46 | 58 | M | No | 10/15/2009 |
| S48 | 54 | F | No | 12/7/2009 |
| S51 | 55 | F | No | 12/7/2009 |
| S52 | 52 | F | No | 12/8/2009 |
| S53 | 57 | F | No | 12/9/2009 |
| S54 | 58 | M | No | 12/10/2009 |
| S55 | 49 | F | No | 12/11/2009 |
| S56 | 55 | M | No | 12/18/2009 |
| S59 | 59 | M | No | 12/18/2009 |
| S201 | 51 | M | No | 12/6/2009 |
| S202 | 54 | M | No | 12/8/2009 |
| S1 | 59 | M | Yes | 10/8/2009 |
| S2 | 59 | F | Yes | 10/14/2009 |
| S3 | 56 | M | Yes | 10/14/2009 |
| S4 | 55 | M | Yes | 10/14/2009 |
| S6 | 52 | F | Yes | 9/4/2009 |
| S7 | 56 | F | Yes | 9/4/2009 |
| S8 | 64 | M | Yes | 10/14/2009 |
| S10 | 55 | F | Yes | 10/29/2009 |
| S21 | 58 | M | Yes | 10/14/2009 |
| S24 | 58 | M | Yes | 10/14/2009 |
| S25 | 59 | F | Yes | 10/14/2009 |
| S26 | 50 | F | Yes | 10/14/2009 |
| S29 | 54 | M | Yes | 10/14/2009 |
| S31 | 52 | F | Yes | 10/15/2009 |
| S32 | 52 | M | Yes | 10/15/2009 |
| S33 | 58 | F | Yes | 10/15/2009 |
| S36 | 48 | F | Yes | 10/15/2009 |
| S38 | 56 | M | Yes | 10/15/2009 |
| S39 | 54 | F | Yes | 10/15/2009 |
| S40 | 55 | M | Yes | 10/15/2009 |
| S41 | 55 | F | Yes | 10/15/2009 |
| S47 | 51 | M | Yes | 10/15/2009 |
| S58 | 61 | M | Yes | 12/18/2009 |
| S22 | 53 | M | Possible | 10/14/2009 |
| S27 | 59 | F | Possible | 10/14/2009 |
| S37 | 64 | M | Possible | 10/15/2009 |

Samples S22, S27 and S37 are not included in the comparison of NJ/76 and Mex/4108/09 in Table 2, Figure 3A-B.
